# Applying the International Classification of Functioning, Disability and Health framework to determine the predictors of falls and fractures in people with osteoarthritis or at high risk of developing osteoarthritis: data from the Osteoarthritis Initiative

**DOI:** 10.1186/s12891-020-3160-5

**Published:** 2020-02-29

**Authors:** Sze-Ee Soh, Anna L. Barker, Renata T. Morello, Ilana N. Ackerman

**Affiliations:** 1 Department of Epidemiology and Preventive Medicine, Monash University, 553 St Kilda Road, Melbourne, Vic 3004 Australia; 2 Department of Physiotherapy, Monash University, 47-49 Moorooduc Highway, Frankston, Vic 3199 Australia; 3 Medibank Private Limited, 720 Bourke Street, Melbourne, Vic 3008 Australia

**Keywords:** Falls, Fractures, Osteoarthritis, Older people

## Abstract

**Background:**

Falls are a major cause of injury and death among older people. Evidence suggests that people with osteoarthritis (OA) are at a higher risk of falls and fall-related injuries including fractures. While studies demonstrate a link between OA and falls, little is known about the pathways that link falls with demographic factors, OA impairments, activity limitations and participation restrictions. The aim of this study was to identify risk factors for falls and fractures among people with OA or at high risk of developing OA using the International Classification of Functioning, Disability and Health (ICF) framework.

**Methods:**

A longitudinal analysis of data from the Osteoarthritis Initiative (OAI) dataset was undertaken. Participants were considered to have OA if they reported they had been diagnosed with knee or hip OA by a medical practitioner. Outcomes were self-reported falls and fractures. Potential predictors were classified using the ICF framework. Poisson regression models were used to determine the risk factors for falls and fractures.

**Results:**

Of the 4796 participants, 2270 (47%) were diagnosed with knee and/or hip OA. A higher proportion of participants with OA reported having had falls (72% vs 63%; *p* < 0.0001) and fractures (17% vs 14%; *p* = 0.012) than those without OA. Personal factors were found to be stronger predictors of falls and fractures compared to OA impairments, activity limitations and participation restrictions in this sample of participants. After adjusting for potential covariates, self-reported history of falls was a significant predictor of both increased falls (incidence rate ratio [IRR] 1.50; 95% confidence interval [CI] 1.40, 4.60) and fracture risk (IRR 1.38; 95% CI 1.13, 1.69).

**Conclusions:**

By applying the ICF framework, we have shown that personal factors were more likely to predict falls and fractures rather than OA impairments, environmental factors, activity limitations and participation restrictions in people with OA or at high risk of developing OA. This highlights the importance of questioning patients about their previous falls and past medical history, and using this information to focus our assessment and clinical decision-making processes.

## Background

Falls are a major cause of injury, hospitalisation and death among older people worldwide [1, 2]. In the United States, it is estimated that approximately 30,000 adults over the age of 65 died as a result of a fall in 2016 [3]. In England, falls have been found to be the ninth highest cause of disability-adjusted life years and the leading cause of injury in 2013 [4]. There is growing evidence that people with osteoarthritis (OA) may be at a higher risk of falls, injurious falls and fall-related injuries including fractures [5–9]. Osteoarthritis is one of the most disabling musculoskeletal conditions among older people with profound impacts on their mobility and ability to work and participate in social roles [10]. Many of the physical impairments associated with OA are also known risk factors for falls such as reduced strength and balance [11, 12]. However, previous studies examining the association between OA and falls have not always considered or conceptualised the pathways that link falls with demographic factors, impairments, activity limitations and participation restrictions [6, 8, 9, 13, 14]. This makes it challenging to draw conclusions about the ways in which a condition such as OA might contribute to a person’s risk of falls and fractures, and to provide relevant clinical recommendations.

The International Classification of Functioning, Disability and Health (ICF) is a theoretical framework that describes the components of health and health-related domains, including for people with chronic conditions such as OA [15]. The multi-perspective approach adopted by the ICF is a useful way to classify the symptoms and outcomes related to OA (Additional file 1, available online), as well as the potential risk factors or predictors of falls and fractures. The aim of this study was to identify the risk factors for falls and fractures using the ICF framework among people with OA or at high risk of developing OA, allowing us to better understand the potential contribution of demographic characteristics, lifestyle behaviours, and OA symptoms and impairments.

## Methods

### Data source and participants

This was a retrospective analysis of de-identified data obtained from the Osteoarthritis Initiative (OAI) database (https://nda.nih.gov/oai), a prospective longitudinal cohort study. The OAI comprises data from 4796 participants aged 45–79 years recruited from four clinical sites in the United States. Details regarding the OAI are described in detail elsewhere [16]. For this current longitudinal analysis, we considered participants to have OA if they reported that they had been diagnosed with hip or knee OA by a medical practitioner (clinical diagnosis with or without radiological evidence) within 12 months of the corresponding data collection period [8]. The non-OA comparator group comprised of participants in the OAI who were at high risk of developing OA but had not yet been diagnosed with the condition. Specific datasets used were 0.2.2, 0.2.3, 1.2.1, 3.2.1, 5.2.1, 6.2.1, 8.2.1 and 10.2.1. Ethics approval was obtained from Monash University Human Research Ethics Committee (MUHREC Project Number 11755).

### Measurement of falls and fractures

All participants in the OAI dataset were asked to self-report if they had a fall and landed on the floor or ground in the last 12 months annually from baseline to year 4, and then at the year 6 and 8 visits. Participants were classified as having fallen if they responded affirmatively to this question at any time point. At the same time intervals, participants were asked if they had been told by a doctor that they fractured a bone. If they responded affirmatively to this question at any time point, they were considered to have a fracture.

### Predictors of falls and fracture risk

**Fig. 1:**
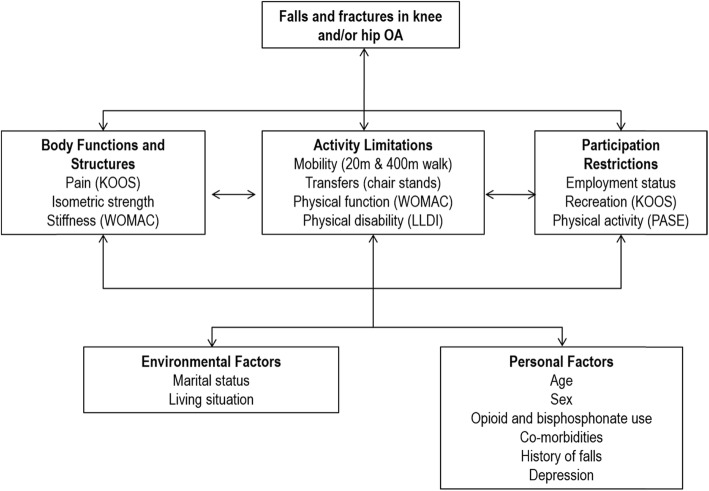
Potential predictors of falls and fractures in people with knee and/or hip OA classified into ICF domains

Demographic, clinical and physiological data were collected from all OAI participants annually from baseline through to year 8. We derived key predictors of falls and fractures from published systematic review evidence on community-dwelling older adults [17]. Potential predictor variables at baseline were extracted from the OAI dataset and mapped to the ICF domains of impairments in body structures and functions, activity limitations, participation restrictions, and environmental and personal factors (Fig. 1). Personal and environmental factors included demographic and clinical characteristics such as age, sex, marital status, living situation, number of comorbidities [18], use of opioids and bisphosphonates, depression [19], and self-reported history of falls. Symptoms associated with knee and/or hip OA such as pain [20], stiffness [21], strength [22, 23], limitations in performing self-care activities of daily living [21] and mobility [24–26] were also examined in relation to falls and fractures. Difficulties relating to recreation [20] and being able to undertake physical activity [27] represent participation restrictions that were also examined as potential predictors of falls and fractures. All predictor variables are presented in Additional file 2 (available online).

### Data analysis

Analysis was performed using Stata/IC15.1 (StataCorp College Station, Texas, USA). Descriptive statistics were used to summarise the characteristics for all participants. Fall and fracture events were also described for all participants in the study. A three-step modelling approach was used to determine the factors associated with increased falls and fracture risk. First, univariate Poisson regression models for falls risk and fracture risk and potential predictors in each ICF domain (Fig. 1) were computed with a robust variance estimator. Second, factors with a moderate association (*p* ≤ 0.10) with the outcome (falls or fracture risk) on univariate analysis were entered into a multivariate model based on their ICF domains and retained if *p* ≤ 0.05 [28]. Highly correlated predictors were identified by the variance inflation factor (VIF) as an indicator of collinearity, and when collinearity was identified (VIF > 2.5) only the variable with the higher R^2^ on univariate analysis was retained for entry into the multivariate model. A final model was computed using all significant variables identified from each ICF domain. We calculated exposure time for each participant from the date of OA diagnosis or study enrolment to the date of survey where the last fall or fracture event was reported. The dependent variable for each model was falls or fracture status following OA diagnosis. Thus, individuals diagnosed with knee or hip OA during the year 8 visit were excluded from analysis because there was no post-diagnosis record of falls and fractures. Interactions between the presence of knee or hip OA and the remaining predictors were also examined.

## Results

### Participant characteristics

Of the 4796 participants in the OAI, 2270 (47%) were diagnosed with knee and/or hip OA by a medical practitioner. Specifically, 1460 participants (30%) reported having been diagnosed with knee OA, while 245 participants (5%) had hip OA and 565 (12%) had both knee and hip OA. The characteristics of participants in the OA and non-OA cohorts were broadly similar (Table 1), although there was a higher percentage of participants with OA using opioid analgesics (*p* < 0.0001) and self-reported a history of falls (*p* < 0.0001). Participants in the OA cohort also reported having more knee pain (*p* < 0.0001), were less physically active (*p* < 0.0001), and had more difficulties performing self-care activities (*p* < 0.0001) and participating in sports and recreational activities (*p* < 0.0001). Analysis of fall and fracture data revealed a significantly higher proportion of fallers among those with knee and/or hip OA compared to those without (*p* < 0.0001). There was also a significantly higher proportion of participants with knee or hip OA who reported having had a fracture (*p* = 0.012).

**Table 1:** Demographic and clinical characteristics of study participants, mapped to the International Classification of Functioning, Disability and Health (ICF) framework

	OA participants (*n* = 2270)^a^		Non-OA participants (*n* = 2526)	
***Fall and fracture outcomes***				
Self-reported fallers, *n* (%)	**1627**	**72**	**1596**	**63**
Self-reported history of fracture, *n* (%)	**382**	**17**	**359**	**14**
***Personal factors***				
Age, mean (SD)	**62.0**	**8.9**	**60.4**	**9.4**
Female, *n* (%)	**1405**	**62**	**1399**	**55**
Number of co-morbid conditions, *n* (%)				
None	**1171**	**73**	**1489**	**79**
One or more	**445**	**28**	**406**	**21**
Depression, mean (SD)	**7.3**	**7.5**	**6.0**	**6.5**
Opioid use, *n* (%)	**218**	**10**	**36**	**1**
Bisphosphonate use, *n* (%)	291	13	285	11
Previous history of falls, *n* (%)	**933**	**41**	**774**	**31**
***Environmental factors***				
Married, *n* (%)	**1466**	**65**	**1712**	**68**
Lived alone, *n* (%)	515	23	540	21
***Impairments in body function and structures (OA impairments)***				
KOOS pain scale, mean (SD)	**72.0**	**20.3**	**84.1**	**16.4**
WOMAC stiffness scale, mean (SD)	**2.5**	**1.8**	**1.5**	**1.5**
Flexor strength (N/kg), mean (SD)	**1.8**	**0.8**	**2.0**	**0.2**
Extensor strength (N/kg), mean (SD)	**4.4**	**1.5**	**4.8**	**1.5**
***Activity limitations***				
WOMAC physical function scale, mean (SD)	**14.6**	**13.2**	**7.4**	**9.9**
Chair stands (stands/seconds, mean (SD)	**0.5**	**0.1**	**0.5**	**0.2**
20 m walk test (m/seconds), mean (SD)	**1.3**	**0.2**	**1.3**	**0.2**
400 m walk test (seconds), mean (SD)	**313.9**	**60.6**	**300.8**	**53.5**
***Participation restrictions***				
Employed, *n* (%)	**1326**	**58**	**1671**	**66**
Physical activity (PASE), mean (SD)	**155.9**	**82.8**	**165.3**	**82.0**
KOOS sport and recreation scale, mean (SD)	**63.8**	**27.1**	**79.4**	**22.7**

*OA* osteoarthritis, *SD* standard deviation, *KOOS* Knee injury and Osteoarthritis Outcome Score, *WOMAC* Western Ontario and McMaster Universities Osteoarthritis Index, *PASE* Physical Activity Scale for the Elderly

Significant results (***p*** **≤ 0.05**) in bold

^a^Of these, 131 participants were diagnosed with OA at Year 8 visit and excluded from further analysis

### Predictors of falls and fracture risk

All personal and environmental factors, OA impairments, activity limitations and participation restrictions examined in the univariate analyses had a moderate association with increased falls risk (*p* ≤ 0.10). However, in the multivariate models for each ICF domain, the only statistically significant personal factors were age, sex, self-reported history of falls and depression (Table 2). Living alone was a significant environmental factor, while extensor strength was the only OA impairment that significantly contributed to falls risk. The time taken to walk 400 m and the ability to participate in sport and recreational activities were also a significant predictor. In the final multivariate model, only personal factors were significantly associated with increased falls risk. The strongest predictors were self-reported history of falls (incidence rate ratio [IRR] 1.50; 95% confidence interval [CI] 1.40, 4.60) and female sex (IRR 1.12; 95% CI 1.04, 1.20). The presence of OA was not an independent predictor of falls risk.

**Table 2:** Univariate and multivariate regression analyses of factors associated with increased falls and fracture risk

	Risk of falls						Risk of fractures					
	Univariate analysis		Multivariate analysis		Final model		Univariate analysis		Multivariate analysis		Final model	
	IRR	95% CI	IRR	95% CI	IRR	95% CI	IRR	95% CI	IRR	95% CI	IRR	95% CI
Presence of OA												
Knee OA only	0.97	0.91, 1.04			0.92	0.85, 1.00	0.86	0.72, 1.02			**0.73**	**0.56, 0.94**
Hip OA only	1.07	0.95, 1.21			1.01	0.88, 1.17	1.05	0.76, 1.45			0.81	0.52, 1.26
Knee and hip OA	1.10	1.02, 1.20			0.92	0.82, 1.03	1.32	1.06, 1.64			0.90	0.62, 1.29
***Personal factors***												
Age	1.00	1.00, 1.01	1.00	1.00, 1.01	1.00	1.00, 1.01	1.01	1.00, 1.02	1.01	1.00, 1.02	1.01	1.00, 1.02
Female	1.17	1.10, 1.24	**1.14**	**1.07, 1.22**	**1.12**	**1.04, 1.20**	1.46	1.25, 1.71	1.21	0.99, 1.46		
Previous history of falls	1.56	1.48, 1.64	**1.51**	**1.42, 1.60**	**1.50**	**1.40, 1.60**	1.49	1.29, 1.72	**1.28**	**1.07, 1.52**	**1.38**	**1.13, 1.69**
Depression	1.01	1.01, 1.01	1.01	1.00, 1.01	1.00	1.00, 1.01	1.02	1.01, 1.03	1.01	1.00, 1.03		
> 1 co-morbid conditions	1.13	1.05, 1.21	1.05	0.98, 1.12			1.50	1.22, 1.76	**1.33**	**1.10, 1.60**	**1.45**	**1.17, 1.81**
Opioid use	1.20	1.08, 1.33	1.07	0.95, 1.21			1.60	1.22, 2.10	1.35	0.96, 1.89		
Bisphosphonate use							1.78	1.48, 2.14	**1.64**	**1.32, 2.05**	**1.78**	**1.40, 2.27**
***Environmental factors***												
Married	0.92	0.87, 0.98	1.00	0.91, 1.09			0.76	0.66, 0.89	0.80	0.64, 1.00		
Lived alone	1.13	1.06, 1.20	**1.13**	**1.02, 1.24**	1.02	0.94, 1.10	1.30	0.10, 1.53	1.08	0.84, 1.38		
***Impairments in body function and structures***												
Knee pain	1.00	0.99, 1.00	1.00	1.00, 1.00			1.00	0.99, 1.00	1.00	0.99, 1.00		
Knee stiffness	1.03	1.01, 1.04	1.02	1.00, 1.05			1.05	1.00, 1.09	1.02	0.96, 1.09		
Flexor strength	0.93	0.89, 0.96					0.97	0.88, 1.0				
Extensor strength	0.96	0.94, 0.98	**0.97**	**0.94, 0.99**	0.99	0.97, 1.02	0.95	0.90, 1.00	0.96	0.90, 1.01		
***Activity limitations***												
Physical function	1.00	1.00, 1.01	1.00	1.00, 1.00			1.00	1.00, 1.01	1.00	0.99, 1.00		
Chair stands	0.72	0.59, 0.87	0.81	0.64, 1.01			0.81	0.46, 1.43				
20 m walk test	0.82	0.73, 0.93	1.03	0.85, 1.25			0.50	0.35, 0.71	**0.58**	**0.34, 0.99**	0.85	0.51, 1.42
400 m walk test	1.00	1.00, 1.00	1.00	1.00, 1.00	1.00	1.00, 1.00	1.00	1.00, 1.00	1.00	1.00, 1.00		
***Participation restrictions***												
Employed	0.90	0.85, 0.95	0.94	0.87, 1.00			0.82	0.71, 0.95	0.89	0.74, 1.06		
Physical activity	1.00	1.00, 1.00	1.00	1.00, 1.00			1.00	1.00, 1.00				
Sport and recreation	1.00	1.00, 1.00	1.00	1.00, 1.00	1.00	1.00, 1.00	1.00	0.99, 1.00	1.00	0.99, 1.00	1.00	0.99, 1.00

*OA* osteoarthritis, *IRR* incident rate ratios, *95% CI* 95% confidence intervals

Final model for falls risk included presence of OA, age, gender, previous history of falls, depression, living situation, extensor strength, mobility limitations (400 m walk test) and difficulties with sport and recreation

Final model for fracture risk included presence of OA, age, previous history of falls, number of co-morbid conditions, bisphosphonate use, mobility limitations (20 m walk test) and difficulties with sport and recreation

Significant results (***p*** **≤ 0.05**) in bold

For the fracture risk analyses, all personal and environmental factors examined had a moderate association on univariate analyses (*p* > 0.10) but in terms of OA impairments, flexor strength was not a significant predictor. Limitations in time taken to stand five times and participation in physical activity were also not associated with fracture risk on univariate analysis. The multivariate models for each ICF domain demonstrated that the significant personal factors that predicted fracture risk were age, self-reported history of falls, having more than one co-morbidity and bisphosphonate use (Table 2). Amongst the activity limitations and participation restrictions examined, a 1 m/s increase in the speed taken to walk 20 m and ability to participate in sport and recreational activities were significantly associated with the risk of fractures. However, when these variables were included in a final multivariate model, only personal factors remained as significant predictors. Bisphosphonate use (IRR 1.78; 95% CI 1.40, 2.27), having more than one co-morbidity (IRR 1.45; 95% CI 1.17, 1.81) and self-reported history of falls (IRR 1.38; 95% CI 1.13, 1.69) were personal factors that contributed significantly to fracture risk. The presence of OA (specifically knee OA) was found to not be associated with increased fracture risk (IRR 0.73; 95% CI 0.56, 0.94).

Sensitivity analyses were conducted to examine the factors associated with the risk of recurrent falls and fractures. Participants who self-reported that they had a fall at two or more time points during the OAI study period were considered to be recurrent fallers. Likewise, participants who reported that they had fractured a bone at more than two time points were considered to have recurrent fractures. Similar to the model for increased falls risk, the strongest predictors for recurrent falls were a self-reported history of falls (IRR 2.09; 95% CI 1.92, 2.27) and female sex (IRR 1.17; 95% CI 1.07, 1.27). Personal factors also remained as significant predictors for having more than one fracture over the OAI study period. Specifically, self-reported history of falls (IRR 2.11; 95% CI 1.63, 2.74) and the use of bisphosphonates (IRR 1.88; 95% CI 1.36, 2.59) and opioids (IRR 1.83; 95% CI 1.18, 2.84) were significantly associated with recurrent fractures. No association was observed between the presence of OA and recurrent falls or fractures. No statistically significant interactions were also observed between the presence of OA and significant predictors for both falls and fracture risk.

## Discussion

This study has applied the ICF framework to classify potential predictors for falls and fractures among people with OA or at high risk of developing OA. We have systematically considered the complexity of OA and adopted a biopsychosocial approach to describe the demographic characteristics, lifestyle behaviours and OA symptoms that may be associated with falls and fractures. Our findings indicate that personal factors are more likely to predict falls and fracture rather than OA impairments, environmental factors, activity limitations and participation restrictions. This has implications for how clinicians can efficiently structure their clinical assessments when evaluating falls risk.

Of the personal factors that were examined, self-reported history of falls was a consistent risk factor for both falls and fractures, It was also associated with an increased risk of having more than one fall or fracture over the study period.. This is consistent with previous research involving community-dwelling older adults which has shown that a history of falls is the single strongest predictor of falls, including recurrent falls [17]. Older adults who have previously fallen or who stumble frequently are two to three times more likely to fall within the next year [17]. In addition, having co-morbidities, using bisphosphonates and using opioids were found to be significant predictors of increased fracture risk. We appreciate that this finding is not necessarily novel given that self-reported history of falls, greater co-morbidity and opioid use are well-established risk factors for future falls [17, 29]. The association between fracture risk and bisphosphonates is also likely due to confounding by indication as bisphosphonates are commonly prescribed to people at increased risk of fractures. Nevertheless, by using the ICF as a framework and taking into account the complexity of OA, we have shown that questioning patients about their previous falls, past medical history and medication profile is an essential component of falls and fracture risk screening even when patients may have other symptoms such as reduced strength and mobility. Given that clinicians have limited time for patient consultations and have multiple competing priorities, these questions can be used to assist clinicians focus their assessments and clinical decision-making processes.

People who are newly diagnosed with knee or hip OA have been shown to be 50% more likely to experience a fall and 85% more likely to experience a fracture 12 months following their diagnosis [8]. Symptomatic knee and hip OA has also been found to be associated with greater odds of future falls [6]. With regard to our study findings that suggested that OA may reduce the risk of fractures, it may be related to participants with OA being less physically active compared to those without the condition. Previous studies have suggested that people with knee and/or hip OA are less likely to fracture because they are less active [30] or are less likely to be in positions where they are at risk of falling and injuring themselves (e.g. walking with a wider base of support or using a gait aid). It is important to note, however, that the association we observed between falls and fracture with physical activity was not statistically significant when other factors were considered. There was also no interaction between physical activity levels and the presence of OA in our study. Although a non-linear association between mobility and falls risk [31] may explain this apparent lack of association, this finding has important clinical implications. Exercise and physical activity are increasingly being recommended to manage OA symptoms and impairments [8, 32]. We therefore need to ensure that an individual’s risk of falling is not inadvertently increased by encouraging them to simply move more, without proper falls risk screening or balance assessment, if indicated.

Although we considered functional impairments and mobility limitations in our analyses, impaired balance was not examined. Balance measures were assessed in the OAI only at the 10-year visit. There is Level 1 evidence demonstrating that falls risk in older people can be reduced by well-designed exercise programs that includes balance activities [33]. Future studies should include static and dynamic balance tests when examining risk factors for falls and fractures in people with OA, to better inform clinical management. This is because the inclusion of exercises that involve a high challenge to balance may be one way in which we can encourage people with OA to move more without increasing their risk of falling [8, 33].

A strength of this study was the large sample of people with falls or fractures and the use of the ICF framework to classify potential risk factors. We have used a biopsychosocial and pathophysiological basis to describe the determinants of falls and fractures including symptoms associated with OA. Nevertheless, some limitations need consideration. As with any secondary data analysis, we were limited to available study variables. The number of falls for each participant was categorised as one, two or three, four or five, and six or more in the OAI dataset. Given the ordinal nature of this variable, we classified those with no falls as a non-faller and those with more than one fall as a faller and binary data were used as an outcome. Additionally, we relied on participants self-reporting falls and fractures at 12-month intervals, which may have resulted in under-reporting of these outcomes [34]. The high proportion of missing data on type and location of fracture [8] also limited our ability to fully describe fractures incurred by the cohort and explore associations between OA and different fracture types. A further limitation was that the predictors of falls and fractures included this analysis were extracted from the OAI study at baseline (i.e. entry into the study) due to the proportion of missing data over time. This limits our ability to determine how changes in a person’s functional status may impact on their falls and fracture risk. Finally, despite including a variety of OA-related impairments, activity limitations and participation restrictions in our analyses, we were unable to explain most of the variance in falls and fracture outcomes. This likely reflects the multitude of complex factors involved, many of which are not directly quantifiable. Our findings therefore highlight the ‘wicked’ nature of falls and the intricate interactions between behavioural, clinical, physical and psychological factors that contribute to a person’s risk of falls and fractures.

## Conclusion

By applying the ICF framework, we have shown that personal factors were most likely to predict the risk of falls and fractures among people with OA or at high risk of developing OA. Specifically, a self-reported history of falls, the presence of co-morbidities and bisphosphonate use were significant contributing factors. This emphasises the importance of clinicians enquiring about previous falls (as well as past medical history and falls-relevant medication use) when screening for falls and fracture risk.

## Supplementary information

**Additional file 1.** Examples of the interactions between the ICF domains for hip and knee OA. Adapted with permission from the World Health Organisation [14].

**Additional file 2.** Predictors of falls and fracture risk.

## Data Availability

The datasets generated and/or analysed during the current study are available from the Osteoarthritis Initiative (OAI) dataset, (https://nda.nih.gov/oai), which is publicly available.

## Abbreviations

CI: Confidence interval
ICF: International Classification of Functioning, Disability and Health
IRR: Incidence rate ratio
KOOS: Knee injury and Osteoarthritis Outcome Score
OA: Osteoarthritis
OAI: Osteoarthritis Initiative
PASE: Physical Activity Scale for the Elderly
VIF: Variance inflation factor
WOMAC: Western Ontario and McMaster Universities Osteoarthritis Index
