# Supplementary material for: Applying the International Classification of Functioning, Disability and Health framework to determine the predictors of falls and fractures in people with osteoarthritis or at high risk of developing osteoarthritis: data from the Osteoarthritis Initiative
Source: BMC Musculoskelet Disord. 2020 Feb 29;21:138. doi: 10.1186/s12891-020-3160-5 (PMC7049177; doi:10.1186/s12891-020-3160-5)
Supplement: Supplementary file 1 — Additional file 1. Examples of the interactions between the ICF domains for hip and knee OA. Adapted with permission from the World Health Organisation [14]. [file 12891_2020_3160_MOESM1_ESM.docx]

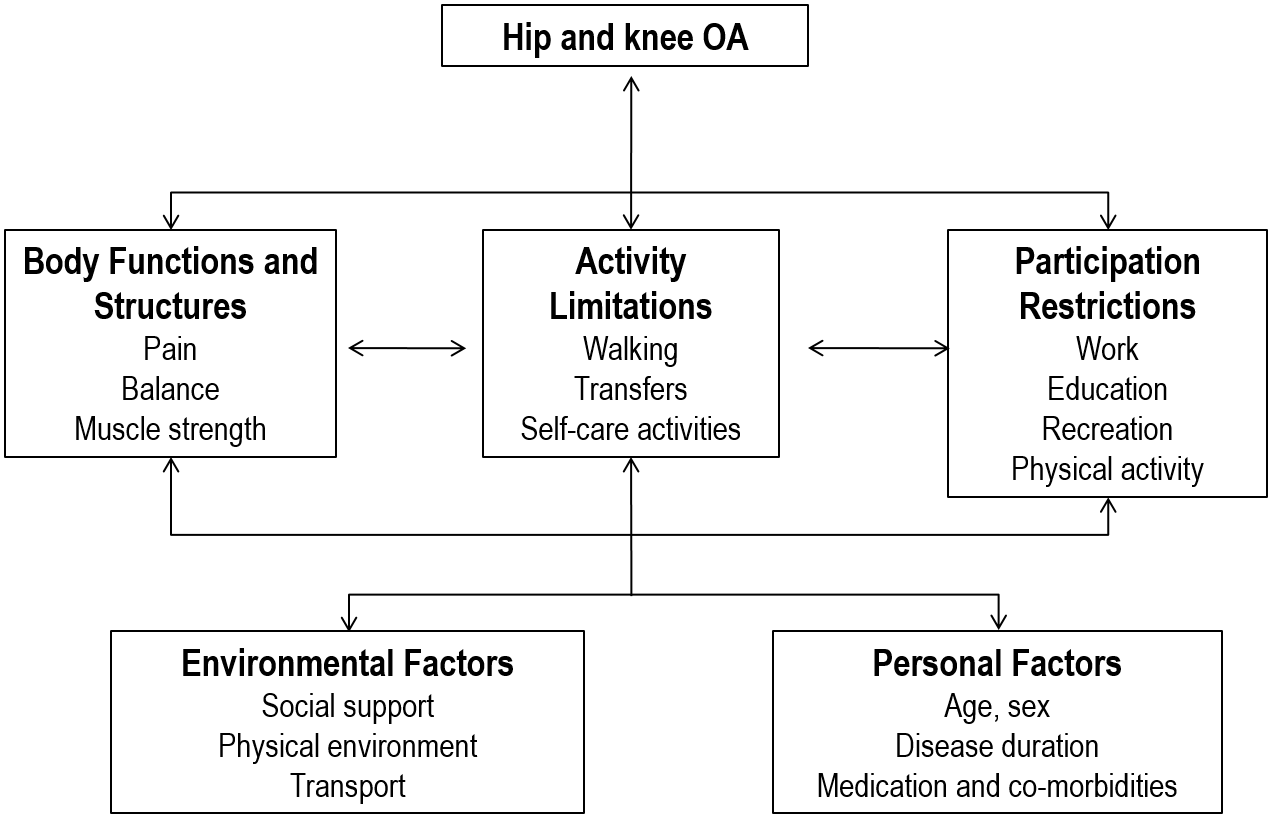


**Additional file 1:** Examples of the interactions between the ICF domains for hip and knee OA. Adapted with permission from the World Health Organisation [15].
