# Supplementary material for: Applying the International Classification of Functioning, Disability and Health framework to determine the predictors of falls and fractures in people with osteoarthritis or at high risk of developing osteoarthritis: data from the Osteoarthritis Initiative
Source: BMC Musculoskelet Disord. 2020 Feb 29;21:138. doi: 10.1186/s12891-020-3160-5 (PMC7049177; doi:10.1186/s12891-020-3160-5)
Supplement: Supplementary file 2 — Additional file 2. Predictors of falls and fracture risk. [file 12891_2020_3160_MOESM2_ESM.docx]

**Additional file 2:** Predictors of falls and fracture risk

| **ICF domain** | **Predictor variable** | **Description** |
| --- | --- | --- |
| Personal factors | Age | Age at enrolment into OAI study |
|  | Sex | Male or female |
|  | Number of co-morbidities | Based on presence or absence of co-morbid conditions using the Charlson Co-morbidity Index Questionnaire [18] |
|  | Opioid use | If narcotic analgesic was used for joint pain during the past 30 days prior to knee and/or hip OA diagnosis |
|  | Bisphosphonates use | If using bisphosphonates (e.g. alendronate, risendronate) at enrolment into OAI study |
|  | Depression | Quantified using the Centre for Epidemiological Studies-Depression (CES-D), higher scores indicate greater symptoms [19] |
|  | History of falls | If participant reported falling in the last 12 months prior to OA diagnosis, or at enrolment into OAI study |
| Environmental factors | Marital status | Married or not married at enrolment into OAI study |
|  | Living situation | Lived alone or not alone at enrolment into OAI study |
| Body functions and structures | Knee pain | Lowest pain score (indicating greater pain) on the KOOS pain scale [20] at entry into OAI study for the two knees of each participant |
|  | Knee stiffness | Highest score (indicating worst stiffness) on the WOMAC stiffness scale [21] of the two knees at entry into OAI study |
|  | Strength | Highest of the three isometric flexor and extensor strength measurements, determined as the maximum of three attempts at 60^0^ knee flexion (Good Strength; Metitur Oy) at entry into the OAI study [22,23]^ξ^ |
| Activity limitations | Self-care activities | Highest score (indicating more severe limitations) on the WOMAC physical function scale [21] of the two knees at entry into OAI study |
|  | Transfers | Assessed using five repetitions of chair stand test, measured in stands per second [24] |
|  | Mobility | Assessed using the 20m walk test, measured in metres per second [25] and the 400m walk test, measured in seconds [26] |
| Participation restrictions | Employment status | Employed or not employed at enrolment into OAI study |
|  | Sport and recreation | Assessed using KOOS function in sport and recreation subscale, lower scores represent more problems in performing these activities [20] |
|  | Physical activity | Measured using the Physical Activity Scale for the Elderly (PASE), higher scores indicate greater activity [27] |

^ξ^Muscle strength data was normalised to body weight, and isometric force measurements (N/kg) were used directly because the OAI muscle strength measurements were taken at an anatomically consistent location [23]
